# Fistula of the Urethra

**Published:** 1835

**Authors:** R. Estep

**Affiliations:** Paris, Ohio; Stark County


**The Western Journal**

OF THE

### MEDICAL AND PHYSICAL SCIENCES.

FOR OCTOBER, NOVEMBER, & DECEMBER 1835.

### ESSAYS AND CASES.

### Art. I.—Fistula of the Urethra.

*Case of Calculi in the Urethra and Bladder—False passage—Imperforate Urethra—Successful Operation.* By Doctor R. Estep, of Paris, Ohio.

Aug. 14th 1834.—Was consulted by David Neidegh, aged 30 years, laboring under symptoms of Calculus, severe paroxysms of which he had been subject to for the last 15 years. *Symptoms present.*—Countenance indicative of extreme pain and anxiety, colorless and rather bloated; pulse feeble and frequent; foul tongue; incessant and painful micturition; in short, a constitution wrecked, and rapidly sinking under the effects of intolerable irritation. On examination of the parts, a calculus, upwards of an inch in length, three fourths of an inch in diameter, of an irregular oval form, is manifest at the bulb of the urethra, where patient is conscious of having felt it, nearly from the commencement of his sufferings. The obstruction occasioned by this stone renders the operation of sounding out of the question; but by the introduction of a finger into the rectum, both tumefaction and crepitation unequivocally proclaim the existence of a plurality of calculi within the cyst.

The operation of lithotomy is proposed and acceded to; but owing to the warmth of the season and the very unpropitious condition of the patient, a course is adopted, with a view to palliate symptoms, and in some measure improve the general health.

Aug. 30th. Patient worse—has discontinued my prescription, and in the vain hope of dissolving the stone, adopted fanciful remedies at the suggestion of ignorant persons. An ulcerated aperture has formed at the anterior extremity of the membranous portion of the urethra, where the stone is lodged, through which most of the urine passes. Patient is clamorous for the operation, which owing to his sinking condition, together with his obstinacy in refusing to pursue a correct course, is finally decided on.

Sept. 4th. Proceeded to patient's residence, prepared to operate, and after administering an injection and a large dose of laudanum—disposing my instruments in order, and binding and supporting the patient in a proper position, took my seat. It may not be improper here to observe, that, owing to the impossibility of sounding, the main operation of cutting into the cyst, could not, as yet, be irrevocably determined on. Notwithstanding, I made my first incision as though I had so determined; i. e. beginning over the stone in the urethra, I continued it down on the left of the raphæ, and terminated at a point between the tuberosity of the ischium and the anus. On removing the stone from the passage, I arose from my seat, and without difficulty introduced the sound, convincing myself and all present of the certainty of stone. Removing the sound and substituting the grooved staff, I resumed my seat, and proceeded to divide the cellular tissue and transversalis muscle, in doing which, two branches (one of considerable magnitude) of the pudic artery were divided, but instantly secured with ligature.

Pursuing with my scalpel the groove of the staff, and the scalpel with the index finger of my left hand, I speedily divided the prostate gland, and found my way into the cyst. Introducing a pair of forceps of the middle size, I grasped and withdrew a very irregular, horned calculus, weighing three drachms eighteen grains, the posterior surface of which was worn smooth by attrition against its fellow, which I removed by a second introduction of the forceps, weighing three drachms. They are both of very angular form; and of a hard, and coarse surface.

Having assured myself that nothing more remained in the cyst, I injected a weak and tepid decoction of flax-seed introduced a catheter, unbound the patient, applied lint and a T bandage, and put him to bed in fine spirits.

The only cutting instruments I employed were, a common pocket-case scalpel, the blade made steady in the scales by a ligature, and a curved bistoury, to enlarge the aperture from within.

Sept. 5th. Patient doing well; passed a comfortable night.

7th. Still improving.

10th. General health good—eats and sleeps well.

13th. A perceptible increase of flesh.

15th. Wound healed, with the exception of that portion occupied by the stone in the urethra, which, on careful examination is found to have lost its original anatomical character, and become a hard cartilaginous sac—scarified the wound with a scalpel.

30th. No improvement in the wound; general health perfectly restored—applied Arg. Nit. and ordered it repeated three times daily.

Oct. 3. Wound as formerly; refreshed the lips; applied two sutures; removed the catheter, and ordered it replaced every alternate day.

7th. Urine follows the sutures; no adhesion; removed the stitches, and applied Ox. Mur. Hyd.

10th. No improvement. From this time on for three months I made trial of every irritating material I could think of, as well as the absence of all of them; Arg. Nit.; Pulv. Canth.; Nit. Acid; Rub. Precip.; Ox. Mur. Hyd.; Ung. Sab.; Lap. Inf. etc.—each, in every degree of activity, was resorted to with much the same results—nothing like granulations or adhesions could be instituted—the lips of the wound skinned over with a kind of mucous membrane, the urine passing in drops through the aperture, without the slightest control of the patient, who is going about in excellent health, but in a most loathsome condition.

Conceiving that time might restore the parts to their original structure, I now determined to let him rest, and after carefully impressing him with the necessity of keeping the passage open by the introduction of a bougie or catheter two or three times a week, took my leave.

May 20th 1835. Notwithstanding the cautions I had given, the patient has, through sheer neglect or indifference, permitted the natural passage to become obliterated at its posterior extremity, to the extent of an inch—the artificial opening remaining as I last saw it. Told the patient I could do nothing for him, unless he would take a room in the village where I could see him at pleasure.

June 9th. Patient having come to town, I proceeded to operate in the following manner. Having supplied myself with a canula seven inches long, furnished with a blunt piston to facilitate its introduction, and a stilette with cutting edges, similar to a trocar, I placed the patient on the side of the bed, his head elevated, and his feet resting on chairs. Introducing the canula with its blunt piston, I applied a cork through the wound to its extremity, and adjusting its direction so as to correspond with the original passage as nearly as possible, I made firm pressure between the end of the canula and the cork—withdrawing the blunt piston and introducing the stilette, I pushed the latter forcibly through into the cork, and by one thrust restored the passage!

The next object, and that which had hitherto baffled every endeavor, was to close the artificial opening, which I find in as unpromising a condition as ever. After contemplating and rejecting every method with which I was acquainted, the following suggested itself to my fancy; and was no sooner suggested than adopted.

Providing myself with two little cylindrical pieces of wood, half an inch longer than the aperture to be closed, five lines in diameter, rounded at the ends and covered with muslin, I made two little perforations through each, seven lines apart, capable of receiving a strong needle and ligature. Arming then a ligature with a needle at each end, I first passed a needle through each of the holes of one cylinder, then through the lips of the wound at a corresponding distance from each other, (the lips being previously cauterized with a red-hot iron,) and finished by passing the needles through the remaining cylinder, over which the ends of the ligature were securely tied.

From this moment, not one drop of urine flowed through the wound, but kindly pursued its course through the newly-made urethra. A perfect cure is the happy result, and the patient has acquired a pretty comfortable control over his discharges, having to urinate but two or three times during the night. The principal object in detailing the above case, is to give publicity to the last (and in this case, only successful) method of procuring a re-union of parts so disinclined to re-unite. The writer feels the utmost confidence, that, it will answer many valuable purposes in practical surgery; and that too, in cases heretofore almost or quite intractable: such as urinary and salivary fistula, hare-lip, lacerated perineum, *et cetera.*

It may be said, that this is but a modification of the old "quilled suture:" be it so, but that modification is conceived to be a very important one. Let it be remembered, that in the application of the quilled suture, the two strans of ligature which literally surround the quills, have a natural and perpetual tendency to separate from each other to the distance of the quills, or in other words, each seeking to become a straight line. Now in any case analogous to the one above described, they would permit the fluid to pass between them, thereby, if it should close one aperture, creating four new ones. Whereas the present, (which I shall denominate the inflexible compress suture,) has the exactly opposite tendency, of closing and compressing the perforation made by the needles, at the same time that it holds the lips of the wound securely in contact.

*Paris, Stark County, O. Oct.* 22, 1835.

